# Analysis of factors associated with brittle response in patients with Parkinson’s disease

**DOI:** 10.1002/acn3.51028

**Published:** 2020-04-30

**Authors:** Yayun Yan, Yanyan Li, Xiufeng Liu, Liyao Zhang, Lu Wang, Ying Chang

**Affiliations:** ^1^ Department of Neurology China‐Japan Union Hospital of Jilin University Changchun China; ^2^ Department of Neurology Weihaiwei People’s Hospital Weihai China

## Abstract

**Introduction:**

The brittle response (BR) in patients with Parkinson’s disease (PD) refers to a special type of levodopa‐induced dyskinesia (LID). This study aimed to describe the clinical characteristics of BR patients and to analyze the associated risk factors.

**Methods:**

A retrospective study was conducted to analyze the data of 97 patients with PD. Patients were divided into a BR group and a non‐brittle response (NBR) group. Demographic and clinical data, motor symptoms, and non‐motor symptoms of the two groups were assessed.

**Results:**

Among 97 PD patients, 11 were in the BR group and 86 were in the NBR group. The proportion of female patients was 72.7% and 38.3%, respectively, in the BR and NBR groups (*P* < 0.05). Compared to NBR patients, BR patients had relatively low body weight, low BMI, long disease duration, high levodopa equivalent daily dosage (LEDD), and high levodopa dose per weight (*P* < 0.05). The BR group had significantly higher scores of UPDRS (II, III, and IV) (*P* < 0.05). But no difference was found in the UPDRS I, emotional state, cognitive status, and accompanied by REM sleep behavior disorder (RBD) (*P*> 0.05). Multivariate logistic regression analysis showed that BR patients had lower body weight and higher levodopa dose per weight.

**Conclusion:**

BR is associated with being female, low body weight, low BMI, long disease duration, high LEDD, and high levodopa dose per weight. Body weight and levodopa dose per body weight are independent risk factors for BR.

## Introduction

PD is a common progressive, neurodegenerative disease, and levodopa is still the gold standard for PD therapy.^1^ However, long‐term use of levodopa can lead to LID. About 40% of patients treated with levodopa for 4–6 years are reported to develop LID.^2^ In fact, the higher the daily dose of levodopa, the greater the risk of developing dyskinesia. However, dyskinesia may appear in only a small number of PD patients even if they are taking a small dose of levodopa at each scheduled time, suggesting that these patients cannot withstand a large dose of levodopa, resulting in poor control of their PD symptoms. The concept of PD “brittle response” (BR) was proposed to describe this phenomenon.^3^, ^4^ However, few reports are found on BR, this study aimed to analyze the risk factors of BR in order to take measures to prevent.

## Subjects and Methods

This study was a retrospective analysis of patient data retrieved from hospital records. The data of a total of 97 patients with PD were extracted from patients’ hospital records. The diagnosis of idiopathic PD was based on the criteria of the International Movement Disorders Society (MDS) in 2015. All patients took levodopa agents regularly and well followed‐up. They were divided into two groups, BR and NBR. BR was defined as dyskinesia (UPDRS, Part IV ≥ 1 point) appearing after taking 100 mg or less of levodopa at the last visit or in the last follow‐up. Patients’ demographic and clinical data were collected, including gender, present age, age of onset, height, body weight, body mass index (BMI), disease duration, UPDRS I to IV scores, the Hoehn &Yahr scale (H & Y scale), current drugs taken and dosages, levodopa equivalent daily dosage (LEDD), and levodopa dose per weight (mg/kg).The instruments used for the survey included the 39‐item Parkinson’s Disease Questionnaire (PDQ‐39) scores, scores of Hamilton Anxiety Rating Scale (HAM‐A), Hamilton Depression Rating Scale (HAM‐D), Mini‐Mental State Examination (MMSE) and Montreal Cognitive Assessment (MoCA), and results of these scales were recorded. All data were analyzed statistically using SPSS 22.0 (SPSS, Chicago, IL, USA). The measurement data of BR and NBR groups were expressed as medians and interquartile range and comparisons between groups using the Wilcoxon rank‐sum test or the chi‐square test or Fisher’s exact test. Multivariate logistic regression analysis was performed to determine the risk factors of BR occurrence. *P* value less than 0.05 was established as statistically significant.

## Results

### Demographic and clinical data

Of the 97 enrolled patients, 11 were in the BR (11.3%) and 86 in the NBR. Female patients in the BR and NBR, respectively, represented 72.7% (8/11) and 38.3% (33/86) (*P* = 0.03). Compared to NBR patients, BR patients had significantly lower body weight (*P* = 0.001), low BMI index (*P* = 0.001), longer disease duration (*P* = 0.013), higher LED (*P* = 0.005), and higher levodopa dose per weight (*P* = 0.001). No statistical differences were found in the age of onset (*P* = 0.56), disease classification (*P* = 0.228), and height (*P* = 0.077) between the two groups. (Table 1).

**Table 1:** Comparison of demographic and clinical data between the brittle response group and the non‐brittle response group.

Items	Brittle response group	Non‐brittle response group	*P*
Gender– (F/M) F(%)	8/3 (72.7%)	33/53 (38.4%)	0.03*
Age of onset–years (>50/≦50) >50(%)	7/4 (63.6%)	62/24 (72.1%)	0.560
BMI index			0.001*
Median	19.5	24	
Interquartile range	17.6–22.5	21.5–25.4	
Height			0.077
Median	165	170	
Interquartile range	160–168	162–173	
Weight			0.001*
Median	53	65	
Interquartile range	48–60	60–75	
Duration of disease			0.013*
Median	8	5	
Interquartile range	6–13	3–9	
LEDD			0.005*
Median	732	387	
Interquartile range	375–900	300–599	
Unit weight of levodopa/benserazide			0.001*
Median	13.8	6.25	
Interquartile range	7.5–19.2	4.2–9.0	
Type of disease (tremor type/tonic type)tremor(%)	10/1 (90.9%)	64/22 (74.4%)	0.228

F, Female; M, Male; LEDD, levodopa equivalent daily dosage.

* Means *P* value less than 0.05 was considered to be statistically significant.

### Motor symptoms

The BR patients had significantly higher scores of UPDRS II, III, IV, H&Y grading than the NBR (the *P* value is between 0.000 and 0.007) (Table 2).

**Table 2:** Comparison of motor symptoms between brittle response group and non‐brittle response group.

Items	Brittle response group	Non‐brittle response group	*P*
UPDRS‐II			0.003*
Median	18	13.5	
Interquartile range	15–24	8–16	
UPDRS‐III			0.009*
Median	39	27.5	
Interquartile range	27–63	18.5–36.25	
UPDRS‐IV			0.000*
Median	11	0	
Interquartile range	8–14	0–4	
H＆Y			0.000*
Median	4	2	
Interquartile range	3–4	1–3	
Freezing			0.007*
Median	2	1	
Interquartile range	1–3	0–1.5	
Symptom fluctuation			0.000*
Median	6	0	
Interquartile range	5–7	0–4	
Dyskinesia			0.000*
Median	5	0	
Interquartile range	3–7	0–0	
Dystonia			0.199
Median	0	0	
Interquartile range	0–1	0–0	

UPDRS, Unified Parkinson Disease Rating Scale; H＆Y, Hoehn–Yahr.

* Means *P* value less than 0.05 was considered to be statistically significant.

### Non‐motor symptoms and quality of life

The BR patients had a significantly higher PDQ‐39 score than the NBR (*P* = 0.001). However, no significant differences were found in the UPDRS I (*P* = 0.276), emotional state (anxiety(*P* = 0.801), depression(*P* = 0.609)), cognitive status (MMSE (*P* = 0.496), MoCA (*P* = 0.711)), and whether it was accompanied by RBD (*P* = 0.162) (Table 3).

**Table 3:** Comparison of quality of life between the brittle response group and non‐brittle response group in non‐motor symptoms.

Items	Brittle response group	Non‐brittle response group	*P*
MMSE			0.496
Median	27	26	
Interquartile range	23–28	24–29	
MoCA			0.711
Median	21	20	
Interquartile range	17–23	17–24	
RBD			0.162
Median	0	0	
Interquartile range	0–12	0–30.25	
HAMD			0.609
Median	6	5	
Interquartile range	3–7	4–8	
HAMA			0.801
Median	5	6	
Interquartile range	3–13	4–8	
UPDRS‐I			0.276
Median	13	11	
Interquartile range	8–17	6.75–13	
PDQ39			0.001*
Median	70	43	
Interquartile range	56–85	24–63	

HAMA, Hamilton Anxiety; HAMD, Hamilton Depression; MMSE, Mini‐Mental State Examination; MoCA, Montreal Cognitive Assessment; PDQ‐39, 39‐item Parkinson’s Disease Questionnaire; RBD, REM sleep behavior disorder; UPDRS, Unified Parkinson Disease Rating Scale.

* Means value less than 0.05 was considered to be statistically significant.

### Binary logistic regression analysis

The risk factors obtained by single factor analysis between BR and NBR were gender, BMI index, weight, duration of disease, LEDD and levodopa dose per weight, the BMI index and LEDD were excluded through multicollinearity assessment, then the other four factor included in the binary logistic regression equation to analyze the independent risk factors for BR. The results showed that body weight (B=−0.111, *P* = 0.025) and levodopa dose per weight (B = 0.206, *P* = 0.008) are independent risk factors for BR. Body weight is a negative factor and levodopa dose per weight is a positive one (Table 4).

**Table 4:** Binary logistic regression analysis of risk factors for BR.

	B	S.E.	Wald	*P*	OR	95% CI for OR	
						lower	Upper
Levodopa dose per weight	0.206	0.078	6.958	0.008*	1.228	1.054	1.431
weight	‐0.111	0.049	5.058	0.025*	0.895	0.813	0.986
Constant	2.572	3.149	0.667	0.414	13.09		

* Means *P* value less than 0.05 was considered to be statistically significant.

## Discussion

Many previous reports on dyskinesia have been published, but descriptions of BR are rare. Therefore, as a special dyskinesia, the discussion of BR in this study may be beneficial to clinicians’ further understanding of BR, early detection, early intervention, and improvement of the quality of life for the affected patients.

LID is a common motor complication of PD. The prevalence of LID depends mainly on patients’ age of onset, disease duration and severity, duration of levodopa treatment, and the dose of levodopa.^5^ The collective evidence suggests that levodopa dose is the more important factor for the development of dyskinesia.^6^ The higher the levodopa, the greater the risk of dyskinesia.^7^ A prospective study of Warren et al.^8^ showed that the risk at < 400 mg/d was 12.1% and at> 600 mg/d was as high as 55.8%. The occurrence of dyskinesia seriously affects patients’ quality of life and can lead to bottlenecks in the treatment of PD patients.

However, in some PD patients, even a small dose of levodopa can cause dyskinesia, which is called the BR. The concept of “brittleness” was first proposed in 1982 when it was described as “the patient has adverse side effects when treated with only a small amount of levodopa”.^4^ In addition to dyskinesia, patients with BR may be in an “open” state of the drug for a very small part of the day, which results in a narrow therapeutic window for drug adjustment, a more complicated treatment option and poor outcome, and patients’ quality of life is seriously affected. No agreement exists at present on the definition of BR. Martinez‐Ramirez et al.^9^ have defined BR as “disabling dyskinesia appearing after a patient has taken 100 mg or less of levodopa at the recent visit or at the last visit before deep brain stimulation (DBS) surgery.” In the present study, BR was defined as dyskinesia (UPDRS IV ≥ 1 point) appearing after a patient has taken 100 mg or less of levodopa at the last visit before DBS surgery or at the last follow‐up for patients who did not undergo surgery. Although dyskinesia is a marker of treatment success, indicating the point at which dopaminergic replacement has been reached and surpassed the therapeutic window, it also suggests that the disease has entered the late stage.

A total of 11 patients with BR (11.3%) were enrolled in this study, which was higher than the 5.5% of BR reported in previous studies^9^ ^.^ The discrepancy can possibly be explained by the relatively small number of cases included in the present study. The results of single‐factor analysis in this study showed that being female, with low body weight, low BMI, long disease duration, LEDD, and higher levodopa dose per weight were all associated with the occurrence of BR. Compared to NBR, BR had a relatively low quality of life and relatively severe motor symptoms of PD. The present study found that the risk factors for BR were similar to those reported for LID.^8^, ^10^ At present, the mechanism of LID is still unclear, although it is believed to be associated with nigrostriatal dopamine depletion^11^ and discontinuous or pulse‐like dopaminergic receptor stimulation,^12^ which leads to the theory of continuous stimulation of the dopamine receptors preventing dyskinesias. Furthermore, LID‐generating mechanisms at the synaptic level show biochemical abnormalities associated with LID in dopaminergic and non‐dopaminergic receptors.^13^ BR is a special manifestation of LID. In fact, not all patients with dyskinesia eventually develop BR. Therefore, we suspect that individuals with BR have their own specificity, as described previously,^14^ and it may involve a certain susceptibility. However, the specific mechanism may be related to the polymorphism of the dopamine receptor gene, which increases the risk of LID.^15^, ^16^

Previous studies have shown that women with PD have a higher risk of dyskinesia,^17^, ^18^ which is compatible with our finding that being female is associated with the risk of BR. It has been reported that the expression of the dopamine receptor D2 (DRD2) gene polymorphism has a certain “gene protective effect,” but the expression of this gene product in female patients seems to be lower,^19^ female patients may have a greater chance of developing BR.

A significant relationship is found between body weight and dyskinesia. In patients with low body weight, the blood drug concentration and the bioavailability of levodopa are relatively high, which shows that body weight correlates negatively with the concentration of levodopa.^20^, ^21^ PD patients often experience weight loss due to excessive consumption during disease progression, which also increases the risk of BR. It has been suggested that after applying the DBS to PD patients, dyskinesia improves and body weight increases significantly.^22^ The improvement of dyskinesia is not only related to the reduced drug dose following the DBS procedure and the anti‐dyskinesia effect of the procedure itself,^23^, ^24^ but is also related to the postoperative increase in body weight. This further demonstrates a certain relationship between low body weight and BR.

Dyskinesia is also associated with the course of the disease. Along with disease progression and the gradual nigrostriatal dopaminergic depletion,^25^ the pulse‐like stimulation of the dopamine receptor by exogenous levodopa causes dyskinesia. Warren et al.^3^ showed that the incidence of dyskinesia increases with the increase in LEDD. In that study, the average LEDD in the BR group was 723.6 mg, far exceeding 600 mg, which indicates that the drug dose was also a risk factor for BR. The dyskinesia is not only associated with the total daily dose of levodopa, but also with its unit weight concentration. Warren et al.^3^ also suggested that the risk of dyskinesia was significantly increased in patients with levodopa dose per weight> 4 mg/kg. In the present study, the average levodopa dose per weight in the BR group was 13.6 mg/kg, far exceeding 4 mg/kg. Therefore, the levodopa dose per weight is also a risk factor for BR. The values obtained in the present study are quite different from the results of other studies, which may be related to the fact that the weight of Western subjects in other studies is generally higher than that of Chinese subjects in the present study. Nevertheless, the age of onset and PD subtype may be important risk factors for dyskinesia.^26^, ^27^ However, the present study did not find a correlation between them and BR.

In the present study, multivariate logistic regression analysis showed that only body weight and levodopa dose per weight were independent risk factors for BR. Previous studies on BR suggested that BR is more commonly seen in women of low weight.^9^ A recent study of dyskinesia found that the levodopa dose per weight of women was higher than that of men.^28^ This is due to the relatively low weight of females, which results in a higher blood concentration of levodopa, making the bioavailability of levodopa relatively high^20^ and increasing the risk of females developing dyskinesia, indicating that gender is not an independent risk factor for the occurrence of the BR. Consistent with the conclusion of the present study, body weight and levodopa blood concentration are the most important independent risk factors of BR.

As a special form of dyskinesia, BR can be resolved by either adjusting the levodopa medication or performing DBS surgery. Regarding the adjustment of medication, we should attempt to reduce levodopa dosage under the premise of improving motor symptoms. The daily dose of levodopa can be controlled to be within 600 mg or even 400 mg, and the levodopa dose per weight can be maintained at 4 mg/kg.^8^ Regarding surgery, 4 of the 11 patients with BR in the present study underwent DBS surgery, and not only the motor symptoms, but BR symptoms also improved significantly, which is consistent with previously reported results.^8^, ^29^ The positive results of undergoing DBS are likely associated with the postoperative weight gain, decreased levodopa dose, and anti‐dyskinesia effect.

There were also deficiencies in this study: the number of cases included in the study was relatively small, and there was no comparative analysis of moderate and severe degrees in patients with a brittle response. The UPDRS III only analyzed the statistics of the medication during the off period and various parts of the motor symptoms were not evaluated separately; the cognitive assessment was roughly evaluated by MMSE and MOCA without stratified analysis, and no specific cognitive domains were studied; this study is not a longitudinal study, and later studies should carry out intense follow‐up and expand the range of study in order to obtain more accurate results.

In conclusion, BR in PD patients is associated with being female, low body weight, low BMI, long disease course, high LEDD, and high levodopa dose per body weight. Body weight and levodopa dose per weight are independent risk factors for BR.

## Conflict of Interests

The authors declare that they have no potential competing interests.

## Authors’ Contributions

YYY and YYL performed the statistical analysis and drafted the manuscript. YYL, LYZ, LW, and XFL collected the clinical materials. YYY and YC double‐checked the statistical analysis and revised the manuscript. All authors approved the final manuscript.

## Ethics approval and consent to participate:

Not applicable.

## Consent for publication

Not applicable.

## Data Availability

The datasets used during the current study are available from the first and corresponding author on reasonable request.
